# A hybrid data envelopment analysis—artificial neural network prediction model for COVID-19 severity in transplant recipients

**DOI:** 10.1007/s10462-021-10008-0

**Published:** 2021-04-23

**Authors:** Ignacio Revuelta, Francisco J. Santos-Arteaga, Enrique Montagud-Marrahi, Pedro Ventura-Aguiar, Debora Di Caprio, Frederic Cofan, David Cucchiari, Vicens Torregrosa, Gaston Julio Piñeiro, Nuria Esforzado, Marta Bodro, Jessica Ugalde-Altamirano, Asuncion Moreno, Josep M. Campistol, Antonio Alcaraz, Beatriu Bayès, Esteban Poch, Federico Oppenheimer, Fritz Diekmann

**Affiliations:** 1 Department of Nephrology and Renal Transplantation, Hospital Clínic, Villarroel 170 (Escala 10 – Planta 5), 08036 Barcelona, Spain; 2 Laboratori Experimental de Nefrologia i Trasplantament (LENIT), Institut d’Investigacions Biomèdiques August Pi i Sunyer (IDIBAPS), Barcelona, Spain; 3 Department of Medicine, University of Barcelona, Barcelona, Spain; 4 Red de Investigación Renal (REDINREN), Madrid, Spain; 5 Faculty of Economics and Management, Free University of Bolzano, Piazza Università 1, 39100 Bolzano, Italy; 6 Department of Economics and Management, University of Trento, Trento, Italy; 7 Department of Infectious Diseases, Hospital Clinic Barcelona, Barcelona, Spain; 8 Institut d’Investigacions Biomèdiques August Pi i Sunyer (IDIBAPS), Barcelona, Spain; 9 Department of Urology, Hospital Clinic Barcelona, Barcelona, Spain

**Keywords:** COVID-19, Kidney transplant, Data envelopment analysis, Artificial neural network, Logistic regression, Random forest

## Abstract

In an overwhelming demand scenario, such as the SARS-CoV-2 pandemic, pressure over health systems may outburst their predicted capacity to deal with such extreme situations. Therefore, in order to successfully face a health emergency, scientific evidence and validated models are needed to provide real-time information that could be applied by any health center, especially for high-risk populations, such as transplant recipients. We have developed a hybrid prediction model whose accuracy relative to several alternative configurations has been validated through a battery of clustering techniques. Using hospital admission data from a cohort of hospitalized transplant patients, our hybrid Data Envelopment Analysis (DEA)—Artificial Neural Network (ANN) model extrapolates the progression towards severe COVID-19 disease with an accuracy of 96.3%, outperforming any competing model, such as logistic regression (65.5%) and random forest (44.8%). In this regard, DEA-ANN allows us to categorize the evolution of patients through the values of the analyses performed at hospital admission. Our prediction model may help guiding COVID-19 management through the identification of key predictors that permit a sustainable management of resources in a patient-centered model.

**Supplementary Information:**

The online version contains supplementary material available at 10.1007/s10462-021-10008-0.

## Introduction

Health systems are designed to meet the daily health care demand of a designated region. But in an overwhelming demand scenario, such as the SARS-CoV-2 pandemic, pressure over health systems may outburst their predicted capacity to deal with such extreme situations (Emanuel 2020). One of the critical points in the context of a pandemic of such magnitude is the potential collapse of the health care system, leading to suboptimal treatment due to a lack of resources and a subsequent increase in mortality. Therefore, in order to successfully face a health emergency, scientific evidence and validated models are needed to provide real-time information that could be used by any health center (Pencina et al. 2020; Wynants 2020a).

Solid organ transplant (SOT) recipients are considered a high-risk population for any infection (Kumar and Ison 2019; Ison and Hirsch 2019; Silva et al. 2020). Preliminary data on SARS-CoV-2 infection shows a mortality as high as 28% (Fernández-Ruiz et al. 2020; Akalin et al. 2020) compared to 7.2% among the general population (Onder et al. 2020). It must be highlighted that fatalities may be attributed to chronic immunosuppression and its associated risks, since these patients are also older and present more comorbidities (Fernández-Ruiz et al. 2020; Akalin et al. 2020; Alberici et al. 2020a; Pereira et al. 2020; The 2019; Guan 2020).

Transplant centers have adapted their policies and developed specific circuits for SOT recipients in the current pandemic scenario (Martino et al. 2020; Boyarsky 2020; Angelico 2020). In particular, strategies for the management of immunosuppression were designed based on pathophysiology and reports of case series, rather than solid studies (Boyarsky 2020; Alberici et al. 2020b).

Predictive models are particularly relevant in situations in which solid evidence is scarce or absent since they may help identifying those patients with high risk of unfavorable outcomes. In the COVID-19 pandemic, they may be of particular benefit for high risk but also for low-volume populations, such as SOT recipients. Although some models are already being developed in kidney transplantation scenarios (Loupy 2019; Aubert 2019), and even in SARS-CoV-2 infection ones (Wynants 2020a; Giordano 2020; Weitz et al. 2020), they are often limited to large cohorts of patients.

The hybrid technique proposed exploits the capacity of Artificial Neural Networks (ANNs) to extrapolate the clinical course of patients using the results of the analyses performed at hospital admission while learning from the categories defined through Data Envelopment Analysis (DEA). This latter method is generally applied in industrial engineering to measure the efficiency of production processes (Misiunas et al. 2016; Ahmadvand and Pishvaee 2018). Its applications in medicine to evaluate the course of patients have overlooked its qualities as a categorization mechanism, a particularly useful feature when dealing with relatively small datasets (Misiunas et al. 2016; Ahmadvand and Pishvaee 2018). The importance of the suggested technique and its main contribution consist in the substantial improvement in the accuracy of the ANN relative to a direct implementation of the latter to the categories defined directly from the output variables.

Therefore, our objective is to develop a predictive model based on the implementation of DEA-ANN to the data retrieved from our cohort of SOT patients with COVID-19 at hospital admission and extrapolate the clinical course of the disease while identifying patients at risk of progressing towards severe disease.

The paper presents the main results obtained from the implementation of our hybrid model to the data retrieved—and analyzed—by the team of doctors. Additional methodological explanations regarding the design of the study, together with the formal definition of the hybrid model and the alternative configurations considered, are provided in the “Appendix” sections.

### Literature review

Physicians tend to distrust the results derived from artificial intelligence models (Bae 2020; Wynants 2020b; Editorial. 2021). This behavior can be intuitively verified through the results obtained in the current paper, where, as we will observe, the machine and deep learning techniques presented do not perform particularly well on their own. Despite these drawbacks, artificial intelligence techniques have been consistently applied to medical settings within the current pandemic scenario. Among the main applications considered, we must highlight their use as early detection mechanisms and monitoring devices (Arora et al. 2020; Rasheed 2020; Vaishya et al. 2020). That is, the use of artificial intelligence techniques in medical environments has mainly focused on disease identification and propagation models (Rashid and Wang 2021). The predictive capacity of these techniques is quite limited, particularly when dealing with small amounts of data and requiring physicians to consider multiple outputs to extrapolate the potential evolution of patients, as is the case in the current paper.

A classification alternative to artificial intelligence models is given by the implementation of multiple criteria decision making techniques. These techniques are usually applied to rank COVID-19 symptoms in terms of their importance and imply a considerable degree of subjectivity in the selection of the weights assigned to the criteria (Alzubaidi et al. 2021). Their extrapolation capacity is also limited. More precisely, their rankings are conditioned by the relative importance assigned to the different decision criteria while lacking an explicit formalization of the actual interactions taking place between the input and output variables (Albahri et al. 2021).

Kidney transplant studies apply machine learning techniques such as random forest (Massie 2020), and artificial intelligence models such as Bayesian networks (Siga 2020) to estimate the relative importance that different factors have on the potential evolution of transplant patients. These techniques remain constrained in their capacity to select several potential output variables simultaneously, imposing a restriction on their ability to extrapolate the actual behavior of patients.

Outside the medical domain, hybrid models defining evaluation indexes combined with machine learning techniques such as random forest have been applied to analyze, for instance, the credit risk of borrowers (Rao et al. 2020a, b). Similarly, DEA has been consistently combined with ANN to generate classification models exploiting the main features of both techniques (Toloo et al. 2015). However, hybrid models combining DEA and ANN are generally based on the efficiency indicators provided by DEA, namely, the $\theta_{o}^{*}$ variables derived from Eq. (1) in the “Appendix” section, a consistent feature that remains mainly unmodified nowadays (Tsolas et al. 2020).

The main problem with this latter approach when applied to a medical context is the fact that physicians do not have an intuitive interpretation for the notion of efficiency. Moreover, imposing the efficiency value as the main selection mechanism implies a substantial loss of information, ignoring the importance of the slack variables introduced in Eq. (2) within the “Appendix” section. Both these drawbacks motivate the design of our slack-based performance index, whose contribution to the identification and classification capacities of different machine learning techniques is summarized in the next section.

## Contribution

The paper balances three very different research areas. First, it provides a precise description of the main analyses performed by the medical team, illustrating their evaluation processes, and defining a benchmark that can be easily recognized by other physicians. Second, it extends the standard analysis of industrial engineers on DEA to generate an index from the slack variables provided by the corresponding optimization model. That is, the use of the slack variables to create a relative performance index that summarizes the potential evolution of patients constitutes a contribution in itself. This index extends the potential applications of DEA beyond standard efficiency considerations, which, in situations such as the one analyzed in the paper, may not be of particular use to physicians. Third, the artificial intelligence techniques employed—and enhanced through the DEA index—provide a general picture of the results to be expected by physicians when dealing with relatively small data sets combining both quantitative discrete and categorical variables. We demonstrate how our hybrid technique performs when data are scarce while requiring basic behavioral guidelines from the simulations so that the model can be understood and applied by physicians under regular circumstances.

The balance among areas has been kept by describing the main medical variables and standard statistical analyses within the main body of the paper while relegating the optimization techniques and other detailed medical exams to the “Appendixes”. In this regard, the results obtained from the hybrid DEA-ANN model and the alternative machine learning techniques and output configurations implemented constitute the main focus of the paper.

The main objective of the manuscript is to illustrate the enhanced identification capacity that results from the design of a hybrid model based on the collaboration of physicians, engineers and computer scientists. As already noted, models combining DEA with artificial intelligence techniques already exist, but the corresponding literature tends to focus on the efficiency scores of the decision making units (Toloo et al. 2015).

From a medical viewpoint, efficiency remains an elusive and complex variable to interpret. Physicians, especially in emergency situations, require instruments based on techniques that—while constrained by a relatively low amount of data and a substantial number of potential output variables—provide them with a set of guidelines describing the potential consequences from an initial evaluation assessment. Artificial intelligence and deep learning models such as neural networks constitute the required techniques. However, their identification and classification capacities are substantially constrained when dealing with few observations and multiple categorical variables. The incorporation of the performance index derived from the implementation of DEA allows us to overcome these drawbacks, leading to a hybrid model whose identification and classification capacities improve substantially upon those of the artificial intelligence techniques applied unilaterally.

## Results

### Patient characteristics

**Fig. 1:**
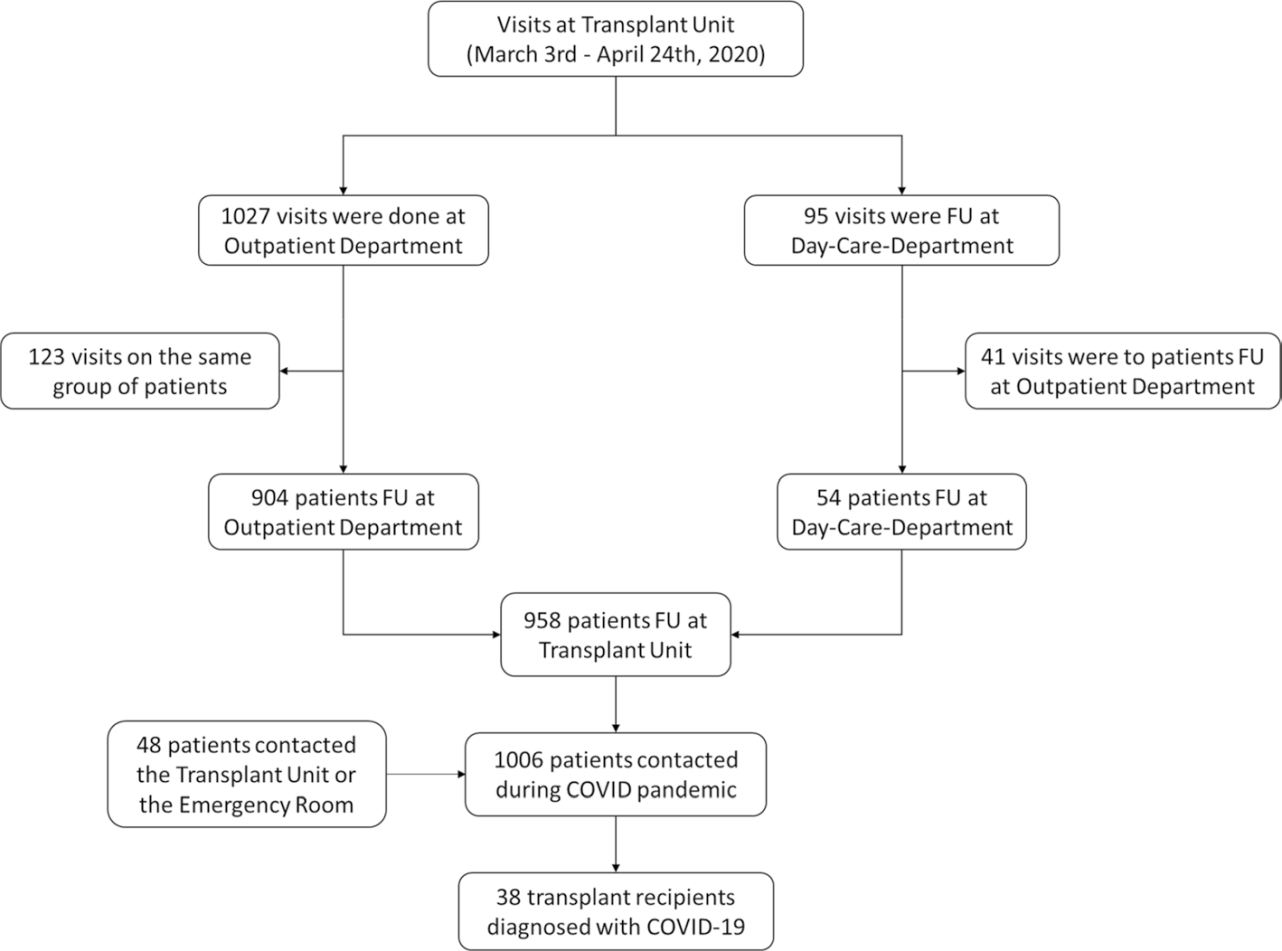
Transplant recipients controlled or attended at the transplant unit. Patients were followed-up from March 3th to April 24th, 2020, as either regular follow-ups, or contacted due to COVID-19 suspicion or other reasons. All patients were asked to report suspicious symptoms. Only patients with high evidence of SARS-CoV-19 infection requiring hospital admission were included in the analysis

During the study period 1006 recipients were visited for regular follow-up or medical issues. Thirty-eight consecutive adult transplant recipients developed confirmed COVID-19 disease requiring hospital admission (Fig. 1), with a mean age of 59 years (range, 33 to 87). Twenty-nine recipients (76.3%) had hypertension, 12 (31.6%) had diabetes mellitus, and 3 (7.9%) had lung comorbidities. Nine patients (23.7%) had been transplanted before, and 16 (42.1%) were under treatment with angiotensin-converting-enzyme inhibitors (ACEI) or angiotensin-receptor blockers (ARB) at hospital admission.

Most recipients consulted with fever (94.7%), cough (60.5%), dyspnea (39.5%) and diarrhea (28.9%), and were admitted after a mean of 7.32 ± 5.87 days from the beginning of symptoms. Thirty-one patients (81.6%) developed pneumonia, sixteen of them (42.1% of the total population) with probable or confirmed bacterial super-infection. Patient characteristics are summarized in Table 1 and Extended Data Table S1.

**Table 1:** Demographic Characteristics at Hospital Admission, infectious and immunosuppressive therapy management, and clinical outcomes in Adult Transplant Recipients Hospitalized with COVID-19

Mean age in years (s.d.) (range)	59.34 (13.26) (33–87)
Male sex, n (%)	23 (60.5)
Comorbidities, n (%)	
Hypertension	29 (76.3)
Diabetes mellitus	12 (31.6)
Chronic obstructive pulmonary disease	3 (7.9)
Previous transplants, n (%)	9 (23.7)
ACEI/ARB treatment, n (%)	16 (42.1)
Main presenting symptom, n (%)	
Fever	36 (94.7)
Cough	23 (60.5)
Dyspnea	15 (39.5)
Diarrhea	11 (28.9)
Positive SARS-CoV-2 test, n (%)	33 (86.68)
Pneumonia, n (%)	
No	7 (18.4)
Unilateral	6 (15.8)
Bilateral	25 (65.8)
Pulmonary Thromboembolism	2 (5.3)
Bacterial co-infection, n (%)	16 (42.1)
Mean time from symptom to hospital admission (s.d.) (range)	7.32 (5.87) (0–21)
COVID-19 intention drug use, n (%)	
Lopinavir/Ritonavir	28 (73.7)
Mena days of Lopinavir/Ritonavir (s.d.) (range)	6.82 (3.49) (1–14)
Hydroxychloroquine	36 (94.7)
Mean days of Hydroxychloroquine (s.d.) (range)	5.58 (1.88) (1–10)
Azithromycin	36 (92.1)
Mean days of Azithromycin (s.d.) (range)	5.52 (1.94) (5–15)
COVID-19 rescue therapy, n (%)	
Tocilizumab	18 (47.4)
Steroid pulse	17 (44.7)
Other drugs (anakinra, interferon, baricitinib)	6 (15.8)
Immunosuppressive approach, n/total (%)	
Calcineurin inhibitor withdraw	30/33 (90.9)
Antimetabolite withdraw	21/22 (95.4)
mTOR inhibitor withdraw	16/16 (100)
Prednisone withdraw	0/32 (0.0)
Stop ACEI/ARB treatment, n/total (%)	12/16 (75.0)
Outcomes, n (%)	
Mean days at hospital (s.d.) (range)	12.02 (6.74) (1–31)
Stay at Intensive Care Unit, n (%)	9 (23.7)
Acute Kidney Injury, n (%)	22 (57.9)
Renal replacement therapy, n/total (%)	2/22 (9.1)
Acute graft rejection, n (%)	1 (2.6)
Graft loss, n (%)	6 (15.4)
Decease-censored graft loss, n/total (%)	1/6 (16.7)
Death, n (%)	5 (13.2)
Discharged from hospital, n (%)	30 (78.9)

Results include all recipients who were hospitalized with COVID-19 from March 3 to April 24, and follow-up till April 27, 2020

*ACEI* Angiotensin-converting-enzyme inhibitors, *ARB* angiotensin-receptor blockers

### Infectious and transplant management

Following our center protocol, lopinavir/ritonavir (73.7%), hydroxychloroquine (94.7%), and azithromycin (92.1%) were given as antiviral therapy. Eighteen recipients (47.4%) required tocilizumab and 17 (44.7%) methylprednisolone pulses. During the follow-up, five patients (13.2%) died with a functioning graft, and 22 (57.9%) developed an acute kidney injury. Thirty patients (78.9%) were discharged from the hospital after a mean of 12.02 ± 6.74 days from admission. Clinical and laboratory data are summarized in Table 1 and Extended Data Table S2.

### Comparative analysis of the poor clinical course of COVID-19

In order to evaluate the primary composite outcomes, we analyzed the clinical worsening profile of recipients in Table 2. Twenty-four recipients (63.2%) fulfilled the criteria for clinical worsening behavior. Cough as a presenting symptom (P = 0.000), pneumonia (P = 0.011), and high levels of lactate dehydrogenase (LDH) (P = 0.031) were admission factors associated with complicated clinical evolutions. Lopinavir/ritonavir and azithromycin were used more frequently in patients displaying a poor evolution. Death-censored graft loss was higher in the composite-group (P = 0.049), absent differences in the management of immunosuppressive therapy. Although a non-significant trend was evidenced in patient and graft survival, no patient died due to COVID-19 in the non-composite-event group. When considering multivariate analyses, cough was the differential risk factor at admission.

**Table 2:** Comparative analysis of composite events

Primary composite outcomes*			
Variable	Composite- events (N = 24)	Non-composite events (N = 14)	*P* value
Mean age in years, (s.d.) (range)	56.53 (13.27)	64.15 (12.14)	0.088
Diabetes mellitus, n (%)	4 (16.7)	8 (57.1)	0.020
Cough as presenting symptom, n (%)	20 (83.3)	3 (21.4)	0.000
Dyspnea, n (%)	12 (50.0)	3 (21.4)	0.097
Pneumonia, n (%)			
No	1 (4.2)	6 (42.9)	0.011
Unilateral	4 (16.7)	2 (14.2)	
Bilateral	19 (79.2)	6 (42.9)	
Mean Lactate dehydrogenase (U/L), (s.d.)	413.91 (119.43)	308.04 (119.49)	0.031
Lopinavir/Ritonavir, n (%)	22 (91.7)	6 (42.9)	0.002
Azithromycin, n (%)	24 (100.0)	11 (78.6)	0.043
Mean days at hospital, (s.d.)	14.55 (6.07)	7.39 (5.47)	0.002
Progression in Radiology findings, n (%)	17 (70.8)	3 (21.4)	0.028
Graft loss, n (%)	6 (25.0)	0 (0.0)	0.049
Death, n (%)	5 (20.8)	0 (0.0)	0.085

^*^Results include all recipients who were hospitalized with COVID-19 from March 3 to April 24, and followed-up till April 27, 2020

χ2 test (or Fisher’s exact test whenever appropriate), Student’s t test, and analysis of variance (Mann–Whitney test)

Nine patients (23.7%) were admitted to the ICU (refer to the Extended Data Table S3). Interestingly, cough, pneumonia (P = 0.039), and high levels of LDH (P = 0.030) prevailed as the differential presenting symptoms.

### Prediction models for COVID-19 severity

**Fig. 2:**
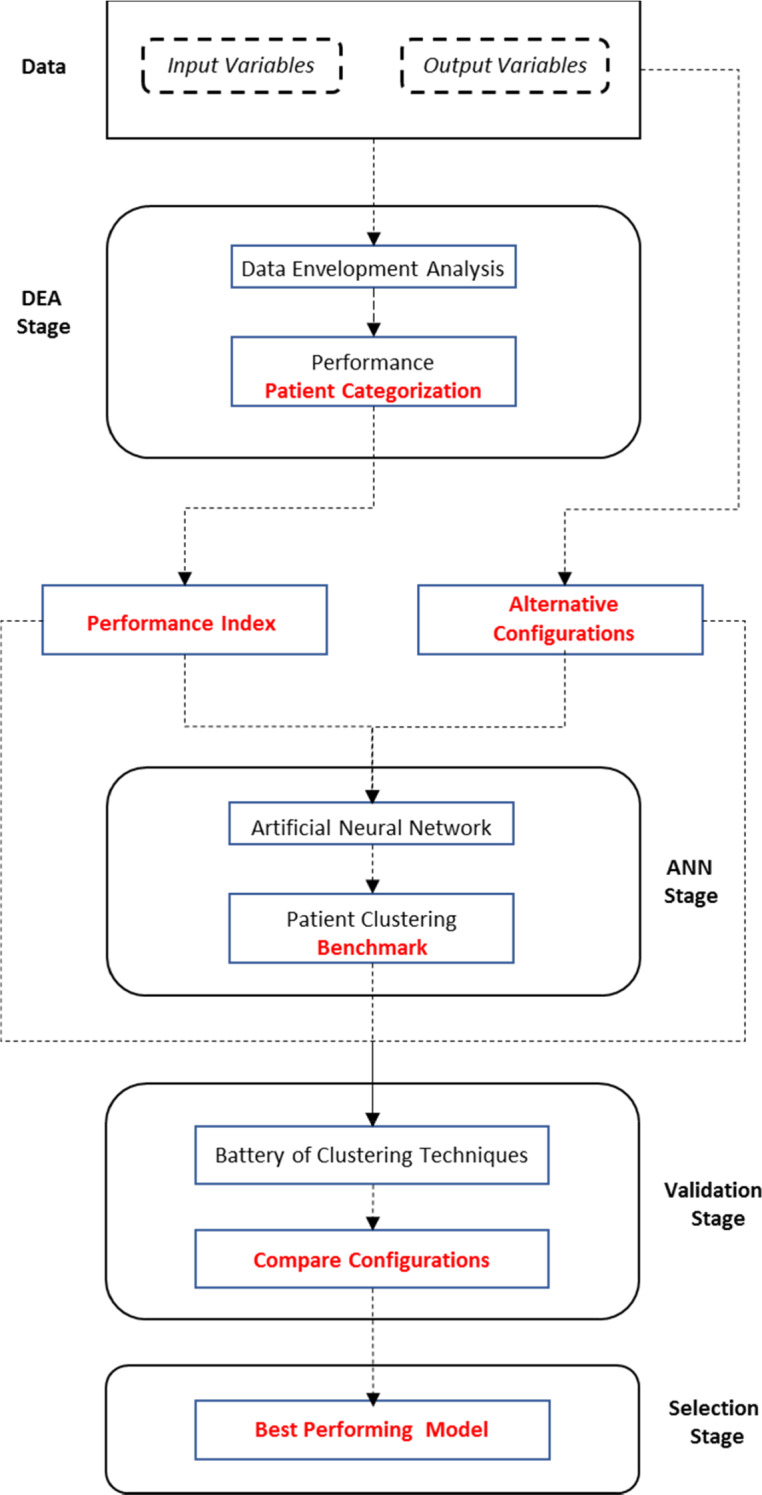
Implementation and evaluation stages of the selection process: hybrid model versus alternative configurations

**Fig. 3:**
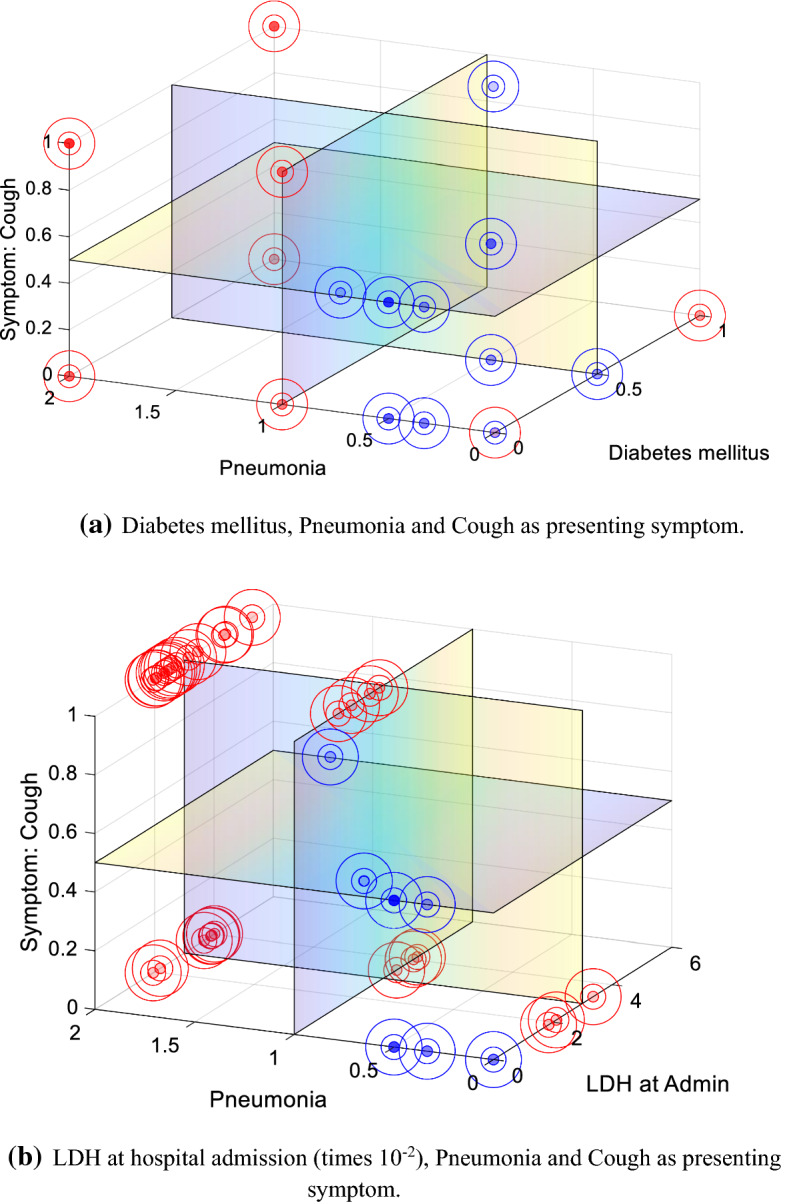
Indexing effect across input variables. The red circles represent the value of the triples described by the data. The blue circles correspond to the index values generated after implementing DEA

**Fig. 4:**
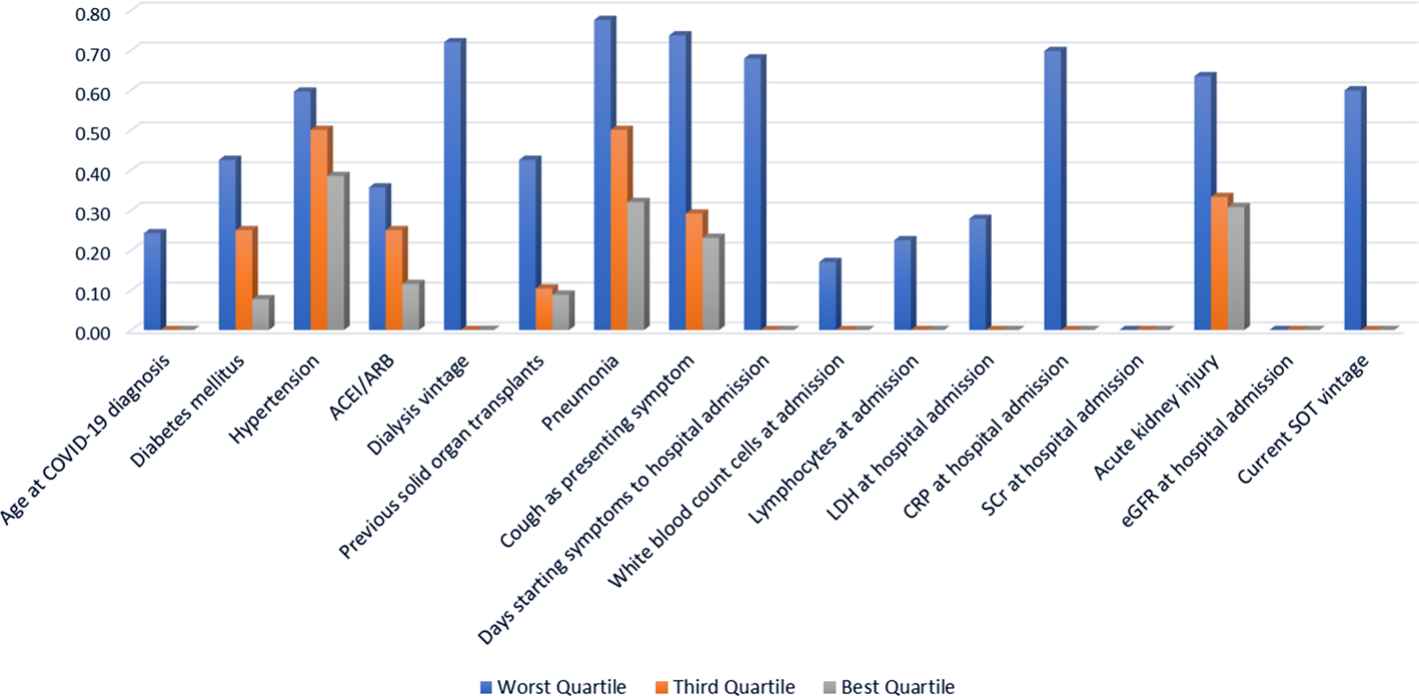
Patient performance across input variables for the different index categories generated using DEA. The index has been normalized within the interval [0, 1]. Higher values represent relatively worse performances

Figure 2 provides a flowchart describing the implementation and evaluation stages of the hybrid model and the alternative configurations described through the current section. The index generated by implementing DEA before being incorporated into the ANN allows us to identify the relative performance of each patient across the variables composing the input set. The intuition validating this result can be inferred from the clustering qualities of the index, already observable in Fig. 3. As a result, we can create average profiles of the patients composing each performance category. Figure 4 provides a concise description of the main variables where patients underperform, with higher values representing relatively worse performances. For instance, note that patients composing the worst quartile exhibit highly suboptimal performances for Cough as presenting symptom, Pneumonia, and Days from starting symptoms to hospital admission. Patients located in the third quartile perform considerably better, with suboptimality arising for Hypertension, Pneumonia, Acute kidney injury and Cough as presenting symptom, describing a similar profile to that of the patients composing the best quartile.

The battery of techniques described in Table 3 has been presented to illustrate the relative performance of the different configurations and their stability. We want to avoid selecting a biased evaluation technique favoring our model while ignoring others where the competing configurations outperform the suggested hybrid. The hybrid DEA model delivers the highest accuracy (96.3%) relative to any of the alternative configurations when considering the ANN that constitutes the main object of analysis. It also outperforms the alternative configurations when considering two well-known techniques such as logistic regression (77.8%) and random forest (codified as bagged trees by the MATLAB interface, 55.6%).

**Table 3:** Accuracy across models and methods

Method	Artificial neural network	Logistic regression	Tree			Linear discriminant	Kernel Naïve Bayes	Support vector machine					
			Fine	Medium	Coarse			Linear	Quadratic	Cubic	Fine Gaussian	Medium Gaussian	Coarse Gaussian
CNF1	55.2	62.1	44.8	44.8	44.8	41.4	27.6	51.7	55.2	51.7	48.3	55.2	48.3
CNF2	69	51.7	58.6^b^	58.6^b^	58.6^b^	27.6	37.9	48.3	51.7	51.7	41.4	48.3	41.4
CNF3	31	65.5	34.5	34.5	34.5	31	27.6	44.8	44.8	41.4	41.4	44.8	41.4
DEA	96.3^a^	77.8^a^	48.1	48.1	48.1	63	33.3	74.1	74.1	77.8^b^	40.7	63	59.3

Method	K-nearest neighbors						Ensemble				
	Fine	Medium	Coarse	Cosine	Cubic	Weighted	Boosted trees	Bagged trees	Subspace discriminant	Subspace KNN	RUSBoosted trees
CNF1	34.5	44.8	48.3	62.1^b^	48.3	48.3	48.3	44.8	55.2	44.8	31
CNF2	37.9	41.4	41.4	51.7	37.9	44.8	41.4	44.8	44.8	31	44.8
CNF3	31	37.9	41.4	41.4	34.5	44.8	41.4	27.6	48.3^b^	27.6	34.5
DEA	70.4	59.3	37	48.1	55.6	70.4	37	55.6^a^	70.4	40.7	59.3

^a^Identifies the highest accuracy score achieved when implementing ANN, logistic regression, and random forest (codified as bagged trees in MATLAB) to the different categorization methods analyzed

^b^Identifies the highest accuracy score achieved by the DEA hybrid and each alternative configuration through the battery of remaining tests

Finally, Table 3 illustrates how the suggested hybrid model tends to outperform the alternative configurations when considering the remaining set of techniques. We have emphasized the highest accuracy score achieved by each configuration through the whole battery of remaining machine learning tests, with the hybrid model performing considerably better than any of the alternatives.

## Discussion

The SARS-CoV-2 infection has become a devastating pandemic worldwide that has pushed all health systems (regardless of their geography) to extreme limits, causing an unprecedented consumption of resources. Due to the potential severity of COVID-19, ICUs have been one of the most needed health devices. In this sense, it is key to determine which patients are candidates to intensive cares, exhaustively evaluating the benefit but also the damage that these intensive measures can cause to patients with extremely high morbidity and mortality.

SOT recipients are more prone to opportunistic infections and worse outcomes from community acquired infections. In patients with the COVID-19 disease, an ICU admission rate as high as 34% (Pereira et al. 2020) and a mortality of 28% have been reported (Fernández-Ruiz et al. 2020; Akalin et al. 2020). These features provide valuable data regarding patient outcomes, but their small cohort sizes and heterogeneity of therapeutic approaches preclude the extrapolation of data into practical decision making.

In the general population, advanced age is associated with a higher risk of ICU admission and mortality (Zhou 2020; Huang 2020). In our composite model, cough, pneumonia, and biochemical parameters at hospital admission, but not age, are correlated with a worse outcome. A confounding factor may emerge when dealing with maintenance immunosuppression. Ageing of the immune system is a well-known process characterized by a defective immune system and increased systemic inflammation (Sato and Yanagita 2019). In patients with chronic kidney disease, these phenomena are accelerated, appearing at younger ages and long-lasting in time (Sato and Yanagita 2019). The association of *inflammageing* (as it is termed) with chronic immunosuppression is a probable explanation for the dissipation of the age effect in our predictive model. Induction immunosuppression, particularly T-cell depleting agents such as thymoglobulin, may increase the risk of disease severity. In an analysis of peripheral lymphocyte populations, Akalin et al. (2020) identified kidney transplant recipients admitted due to COVID19 to have lower CD3, CD4, and CD8 counts than those reported in the general population (Gautret 2020).

Most SOT recipients receive prednisone as their maintenance immunosuppression therapy. Steroids have been described to reduce pathogen clearance and increase the viral shedding of SARS-CoV2 if administered early in the course of the disease (Cao 2020). Hence, most organizations advise against its routine administration in patients with COVID-19 (except if indicated for another reason, such as asthma or acute respiratory distress syndrome) (Luo 2020; Cortegiani et al. 2020; Fontana et al. 2020). Interestingly, steroids, either as monotherapy or in combination with CNI’s, were maintained in most reported series (Akalin et al. 2020; Alberici et al. 2020a; Pereira et al. 2020) and management protocols (Alberici et al. 2020b).

Following the policy of our center, we use tocilizumab and steroids as rescue therapy in patients with poor clinical outcomes despite an initial treatment with lopinavir/ritonavir, hydroxychloroquine, and azithromycin. However, up to now, no treatment has proven to be able to modify the natural history of SARS-CoV-2 infection in a randomized control trial, the evidence supporting the potential benefit of these drugs being preliminary and, sometimes, controversial—even more in SOT recipients (Gautret 2020; Cao 2020; Luo 2020; Cortegiani et al. 2020; Fontana et al. 2020). There are several reports of tocilizumab being used among SOT recipients with COVID19 (Akalin et al. 2020; Alberici et al. 2020a; Fontana et al. 2020), but its benefits are hard to extrapolate, in part due to its use as a second-line treatment, hence only in patients with moderate-severe disease. On the other hand, the use of tocilizumab translates into a good predictor of disease progression and severity. In this study, we were able to identify non-traditional risk factors for disease severity using tocilizumab in a composite model of worse outcomes.

In previous studies, cough has been found with relatively greater frequency in patients with poor outcomes, although, to this date, said symptom has not been included in predictive models to identify those patients with worse prognosis and a high likelihood of being admitted to the ICU (Wynants 2020a; Alberici et al. 2020a; Guan 2020; Zhou 2020). Herein, we have illustrated that the presence of cough at COVID-19 diagnosis constitutes a factor significantly related to the composite outcome of clinical progression and need for intensive care. Analytically, the usefulness of LDH and C-reactive protein (CRP) as markers of more severe forms of COVID-19 has been previously described (Giordano 2020; Zhou 2020). Therefore, some predictive models have used these parameters to identify forms of COVID-19 with a poor prognosis (Wynants 2020a). Similarly, in this study, LDH values at hospital admission have been directly associated with the composite outcome of poor prognosis and a higher probability of ICU admittance.

### Enhancing the accuracy of machine learning techniques

The purpose of implementing a combination of DEA with an ANN is threefold. First, the optimization process on which DEA is built allows us to identify the performance of each patient regarding every analysis-related variable considered. Second, the corresponding slack values are used to design an index indicating the relative performance of each patient when considering several outputs simultaneously. Standard artificial intelligence models are generally constrained by one output variable distributed across different categories, though slightly more complex combinatorial environments may be defined. Finally, the index obtained can be used to orientate the ANN, which would otherwise omit the efficiency interactions existing across variables, leading to a relatively poorer performance.

The performance index has been generated by normalizing the slack values retrieved from DEA relative to the initial ones, being these either quantitative discrete or categorical, and adding up the results across all the input variables. Quantitative discrete variables have been normalized dividing the slack value obtained from DEA by the original value of the variable. The normalization of categorical variables requires dividing the sum of the initial value and the slack obtained from DEA by the sum of the initial value and the maximum value of the category. The final slack-based efficiency sum per patient has been normalized relative to the highest value obtained, implying that higher values of the index correspond to relatively worse performances.

One of the most remarkable features of the hybrid DEA-ANN model is its significant prediction accuracy in environments characterized by relatively low numbers of observations. In addition, it provides a reliable framework of analysis for data structures where multiple input and output variables must be evaluated simultaneously.

When considering machine learning techniques, logistic regression (Bagley et al. 2001) and random forest (Cafri et al. 2018) are among the most commonly used ones in the medical literature. Despite their general applicability, their accuracy tends to decrease when dealing with small data samples, as is the case in the current analysis. We have illustrated how the suggested model (96.3% accuracy) improves substantially upon the best performance of both techniques under any alternative configuration (65.5% for logistic regression and 44.8% for random forest). In addition, the accuracy of both techniques has also been improved when the index generated through DEA is used to define the corresponding output categories (logistic regression achieves an accuracy of 85.2% with the second DEA configuration defined in Table S6, while random forest reaches 55.6%).

### Comparing ANN and DEA-ANN

We start by noting that a common shortcoming of the machine and deep learning techniques implemented throughout the paper is given by their limited capacity to incorporate multiple output categories into the analysis, with the logistic regression representing a limit case. This constitutes a considerable constraint whenever physicians need to consider multiple output variables and their potential interactions. The capacity of these models to identify patterns when directly implementing the categories defined by the different output combinations is also quite limited. We present below the confusion matrices obtained when applying the ANN and the DEA-ANN ranking categorization to the different output categories described in the study. A basic interpretation of the confusion matrices described through this section is provided in Table 4.

**Table 4:** Description of the entries composing a confusion matrix

	Target class		
	1	2	
*Output class*			
1	True positive	False positive	Precision False discovery rate
2	False negative	True negative	Negative predictive value False omission rate
	Sensitivity False negative rate	Specificity False positive rate	Percent of correctly classified cases Percent of misclassified cases

**Fig. 5:**
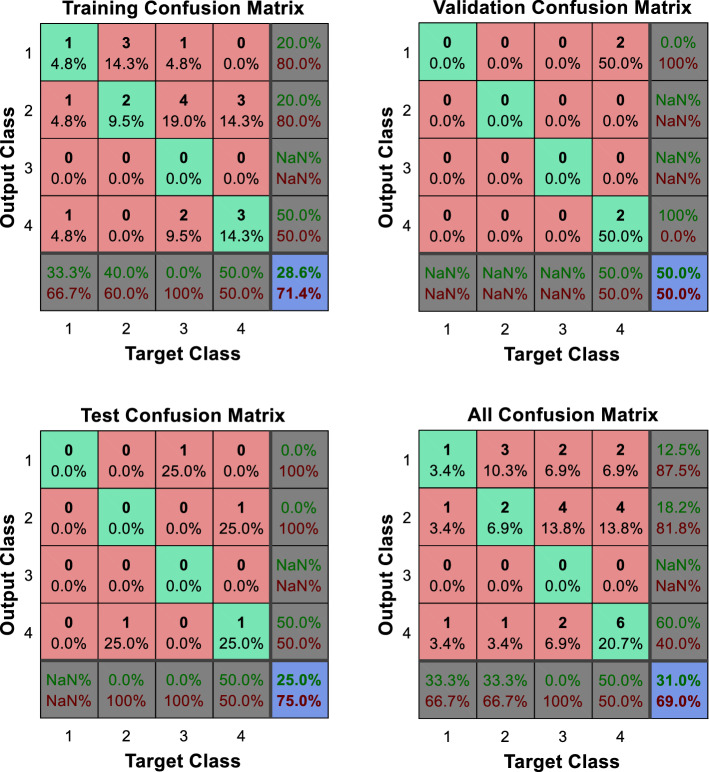
Quartile setting. CNF3 configuration

**Fig. 6:**
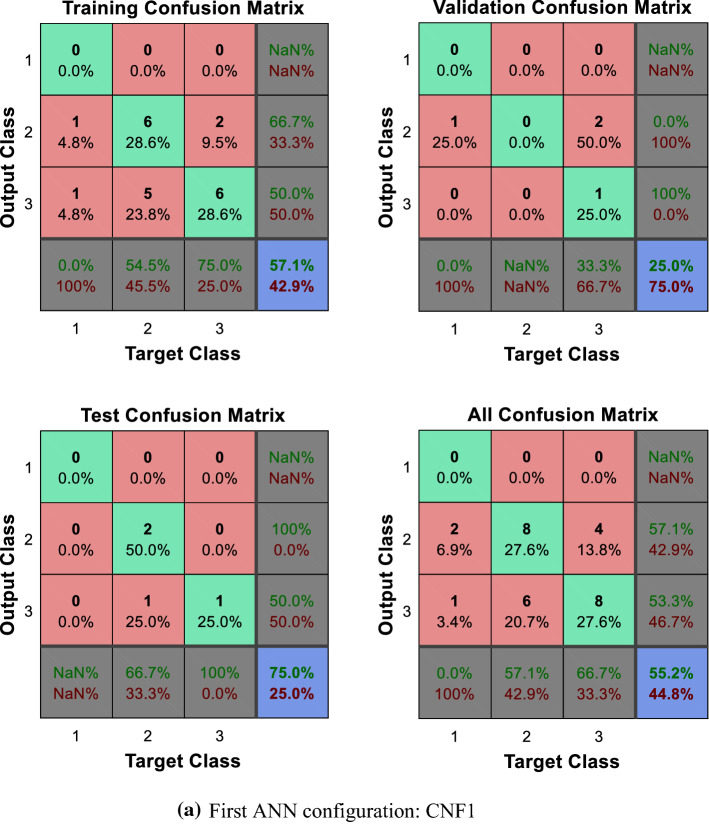

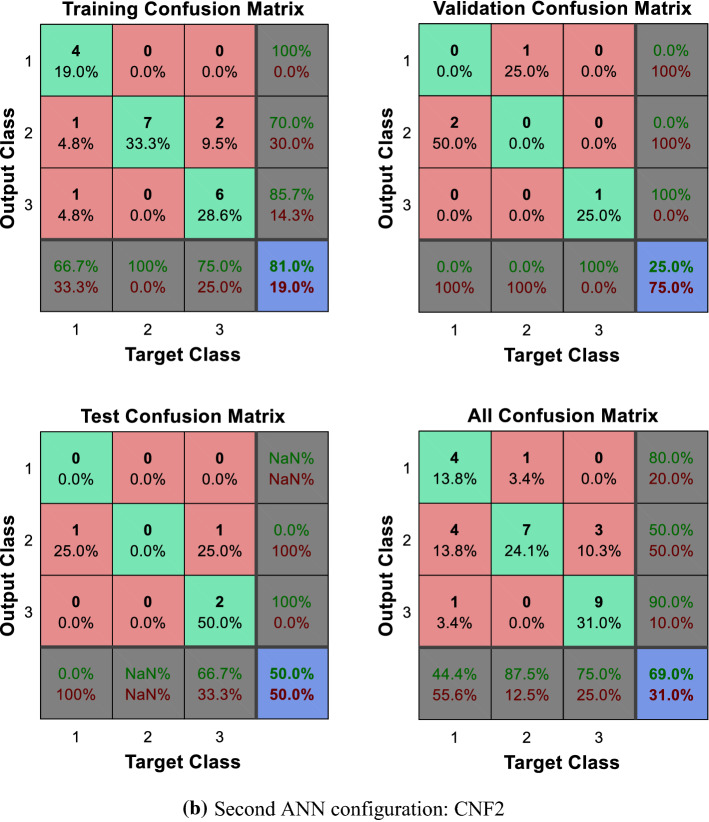

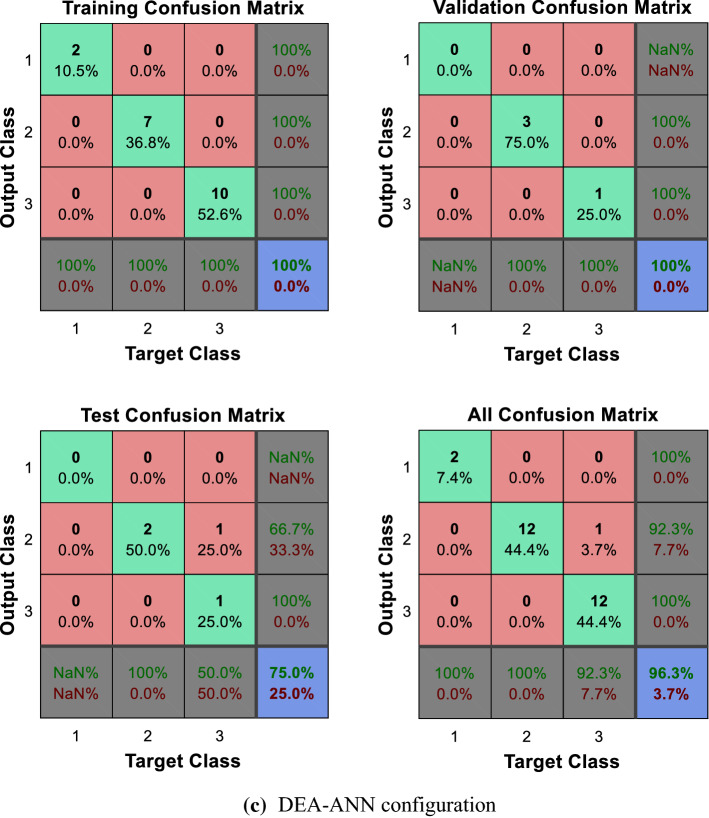
Tercile setting.

As can be observed in Figs. 5 and 6a, b, the direct implementation of the ANN to the different categorization scenarios described, which encompass all the potential combinations of the composite primary outcomes defined by the physicians, does not produce significant results. Note, in particular, the difficulties faced by the ANN to identify those patients located in the worst categories, namely, those displaying relatively poorer output values. The enhanced identification and classification capacities of the DEA-ANN hybrid model are illustrated in Fig. 6c. A similar intuition based on the same type of results follows from the alternative evaluation scenario described in the “Appendix” section, where death and days spent in intensive care are taken as outputs.

## Conclusions

Prediction models are needed to counteract the controversies in data and lack of information in specific populations under the current SARS-CoV-19 pandemic. The prediction of a worse clinical course in high-risk populations facing a sanitary emergency becomes a feasible option with the hybrid DEA-ANN model introduced through the paper. As shown in the study with kidney transplant recipients, cough, pneumonia, and LDH at admission constitute key risk factors for a more severe course, a profile validated and extended through the slacks obtained via DEA. The prediction accuracy of the DEA-ANN model peaks at 96.3%, while the implementation of the ANN to configurations directly based on the value of the output variables achieves a maximum of 69%. A consistent improvement in the prediction accuracy of different machine learning techniques has also been observed when the output categorization process is determined by DEA.

The capacity of DEA to synthesize the behavior of complex processes through its slack variables enhances the ability of the ANN to identify the main relationships existing among the characteristics defining the alternatives. In this regard, one of the main contributions of our hybrid model is the improvement in the predictive power of different machine learning techniques when dealing with few data, a particularly important problem in developing countries, whose hospitals are generally constrained to work with small datasets. Being able to extrapolate the potential evolution of patients should improve the efficiency of patient management and resource allocation processes across hospitals, a particularly important feature in emergency situations.

As can be inferred from the empirical results presented, a drawback of the proposed hybrid model stems from the actual definition of the quartiles, particularly in settings where few observations may result in an empty quartile. Clearly, the model is still able to perform properly and enhance the identification capacity of the ANN, but physicians may have to consider adjusting the distribution of patients across quartiles or terciles, decreasing their confidence in the validity of the results obtained. On the other hand, the model provides a complete profile of the patients composing each quartile category, allowing for a direct evaluation of their relative performances and the potential behavior of the corresponding output variables.

Future research should aim at incorporating dynamic DEA structures in the generation of the index so as to account for complex interactions across input and output categories within a simple framework. Moreover, incorporating the subjective beliefs of physicians into the design of the model would modify its performance but allow for direct interactions with the users, improving its applicability as a decision support system. In this regard, different weights could be assigned to the input and output variables, integrating techniques such as the best–worst ranking method within the current hybrid DEA-ANN framework.

### Supplementary Information

Below is the link to the electronic supplementary material.

Supplementary file1 (DOCX 237 kb)

## Data Availability

The data set supporting the findings of this study are available from the corresponding author (I.R.) upon reasonable request.

## Appendix: Methodology

### Patients and study design

We conducted a retrospective observational study including all kidney transplant recipients admitted due to a SARS-CoV-2 infection between March 3, 2020 and April 24, 2020 at our center. The study was conducted following the approval by the local Institutional Ethical Committee.

#### Diagnosis of covid-19

Infection by SARS-CoV2 was diagnosed by a positive result in RT-PCR tests from a nasopharyngeal swab. However, due to laboratory constrains and the limited diagnostic sensitivity of this technique (Worl Health Organization 2019), since April 2020 the diagnosis of COVID-19 was established by clinical criteria, according to presenting symptoms (presence of respiratory and/or gastrointestinal symptoms with fever), analytical (especially C-reactive protein [CRP], ferritin and lactate dehydrogenase [LDH] increase, lymphopenia), and radiological findings (unilateral or bilateral interstitial pneumonia). In doubtful cases, diagnosis was confirmed by nasopharyngeal swab RT-PCR and/or CT-scan findings.

Nasopharyngeal and oropharyngeal swabs were collected following the Centers for Disease Control and Prevention guidelines, namely, in 3 mL viral transport media and RNA extraction followed by real-time RT-PCR. All sample aliquoting and mixing was performed with an automated Hamilton STARlet 8-channel liquid handler and the assay plates were scanned using a COBAS 6800 RT-PCR system (Roche Diagnostics GmbH, Mannheim, Germany).

#### Patient management

According to center policy, the presence of one or more of the following were considered criteria for hospital admission in patients with a COVID-19 diagnosis: respiratory rate ≥ 20 bpm, baseline SO_2_ < 95%, unilateral or bilateral pneumonia on chest radiography, and/or significant comorbidities (active hematologic disease, neutropenia in patients with active neoplastic disease, poorly controlled HIV infection, a solid organ transplant in the previous 12 months, or recent treatment for acute graft rejection). The criteria for admission to an intensive care unit (ICU) included respiratory deterioration with the need for high doses of oxygen (FiO2 ≥ 60%) and/or PaO2/FiO2 < 300.

During hospital admission, analytical monitoring was performed every two days (daily in severe patients) with serum creatinine (SCr), CRP, procalcitonin, ferritin, LDH, ultrasensitive troponin I (US-TnI), blood count (including lymphocyte count), and serum ionogram (sodium, potassium, calcium, and magnesium). Radiologically, at least two chest radiographs were performed weekly, or every 48 h in severe patients or those with a clinical worsening. Due to the potential QT prolongation and arrhythmic events associated with treatments with lopinavir/ritonavir, hydroxychloroquine, and azithromycin, patients underwent an electrocardiogram every 48 h during treatment in order to identify potential conduction disturbances associated with these drugs.

#### COVID-19 treatment protocol

According to center policy, admitted patients were treated (except contraindications) with a triple combination therapy of lopinavir/ritonavir (200/50 mg/12 h), hydroxychloroquine (400 mg/12 h on the first day, then 200 mg/12 h for 4 more days, except for patients with an estimated glomerular filtration rate [eGFR] < 10 mL/min/1.72m^2^, in which case the dose was 200 mg/day), and azithromycin (500 mg/24 h the first day and then 250 mg/24 h for 4 days). Those patients who despite this treatment presented a clinical worsening with evidence of increase of acute phase reactants (CRP and ferritin) were treated with tocilizumab in a single dose (400 mg if < 75 kg, and 600 mg otherwise). If 24 h after the administration of tocilizumab there was no improvement (or whenever tocilizumab was not available), 3 pulses of methylprednisolone 250 mg/day were administered. Finally, in the absence of clinical improvement, anakinra (200 mg/12 h the first day, and 200 mg/24 h for two more days), interferon, or baracitinib were used. Demonstrated and active bacterial infection was considered a contraindication for the use of tocilizumab (and other immunosuppressants administration).

In addition to antiviral treatment, all patients received prophylactic heparin due to the high risk of COVID-19 associated thrombosis (40 mg/d of enoxaparin), which was maintained until hospital discharge. Those with elevated d-dimer (> 3000 ng/mL) received enoxaparin at a dose of 1 mg/Kg/24 h, and those with confirmed pulmonary thromboembolism received enoxaparin at anticoagulant doses (1 mg/kg/12 h, or 1 mg/kg/24 h if eGFR < 30 mL/min/1.72m^2^) planned to continue for 3 months.

#### Management of immunosuppressive treatment

Due to the potential severity of SARS-CoV-2 infection, mycophenolate and mTOR inhibitor were temporarily withdrawn in all admitted transplant recipients with COVID-19. Furthermore, in severe patients or those starting treatment with lopinavir/ritonavir, the calcineurin inhibitor (CNI) was also temporarily discontinued due to the potential and significant drug interaction with lopinavir/ritonavir. In these cases, maintenance immunosuppression consisted of prednisone in monotherapy (20 mg/day) until COVID-19 resolution, at which time CNI was reinitiated at reduced doses.

#### Study assessments

The primary study outcome consists of the set of potential combinations defined through the events representing a worse course of the patients (namely, ICU needs, Tocilizumab, or pulse of steroids use). We chose a composite primary outcome consisting of a broad definition of worse events for the therapeutic approach until a worse course of the disease was detected.

#### Statistical analysis

Parametric and non-parametric tests were conducted conditioned by the variables being analyzed. Univariate and multivariate binary logistic regression analyses have been performed for the primary composite outcomes and ICU admission. All statistical tests have been conducted within a 95% confidence interval and a P value < 0.05 is considered significant. SPSS v.25 software (SPSS inc, Chicago, IL, US) has been used to perform the statistical analyses.

### DEA–ANN: definition, alternative configurations, and evaluation techniques

The main objective of the hybrid model is to extrapolate the potential course of patients based on the analyses performed at admission. Thus, we studied the effects that a set of 17 input variables has on a group of 3 potential outputs. The input variables used to generate the profiles of the patients and the outputs defining the corresponding treatments are described in Table S4. Note that the output variables correspond to those identified by the physicians as defining the worse composite outcome. Similarly, the input variables include all those identified as significant in previous studies, as described through the main body of the paper, together with those that the physicians considered to be important risk factors among SOT patients.

The main model consists of two specific but interrelated phases. An optimization technique named output-oriented Data Environmental Analysis (DEA) is used to analyze multiple input and output variables simultaneously and identify the magnitude of those where each patient underperforms relative to the benchmark set (Zhu 2014). The resulting values are used to design a performance index defining the output categories on which the Artificial Neural Network (ANN) will be trained. A primer on DEA is provided below. In the second phase, we implement a two-layer feed-forward network with sigmoid hidden and softmax output neurons to cluster patients across the DEA-generated categories. The network is trained using scaled conjugate gradient backpropagation (Bishop 2006).

#### A primer on data envelopment analysis

DEA is generally applied to medical environments when analyzing the performance of facilities and hospitals. Its implementation to evaluate the evolution of patients has been highly limited. Only two papers in the operational research literature follow a path similar to ours. The first one (Misiunas et al. 2016) applies DEA to identify the most efficient patients when dealing with large amounts of data that must be analyzed through an ANN. The second one (Ahmadvand and Pishvaee 2018) focuses on the performance of a fuzzy framework that incorporates data regarding the behavior of both patients and different logistic factors.

The set of variables defining a standard output-oriented DEA structure is described below. The intuition behind the selection of an output-oriented framework is related to the medical interpretation of the model. The output variables will be defined so that increments in their value constitute a main objective of the optimization model. That is, one of the main features of the analysis consists of adapting the medical variables so that the DEA framework aligns with the main objectives of the physicians and provides results that can be easily interpreted.

More precisely, DEA models can be divided in two main categories in terms of orientation, namely, input and output oriented. A DEA model is input-oriented when the inputs are minimized while keeping the outputs at their current levels. Similarly, an output-oriented DEA model maximizes outputs while keeping inputs at their original levels. The aim of our approach is to extrapolate the potential evolution of patients—as described by the behavior of the output variables within the sample group being analyzed—given their initial analyses. This evolution is determined by the relative value of the input variables while considering the behavior of patients through their different output profiles. Thus, since the inputs cannot be modified to improve the profiles of the patients, as an input-oriented approach would require, we focus on the best potential performance of patients and the subsequent constraints imposed by their inputs to perform accordingly.

We consider a set of $n$ patients subject to $m$ different analyses at the hospital admission stage (input variables), $x_{ij}$, $i = 1,...,m$, $j = 1,...,n$, that lead to $s$ potential outcomes (output variables), $y_{rj}$, $r = 1,...,s$, $j = 1,...,n$. That is, the rows of the resulting $n \times (m + s)$ matrix represent the patients whose inputs and outputs are described in the corresponding columns. The DEA model defining the performance score of ${\text{Patient}}_{O}$ is given by:

1 $$\begin{aligned} & \max \,\,\,\,\theta_{o} \\ & {\text{subject}}\;{\text{to}} \\ & \sum\limits_{j = 1}^{n} {\lambda_{j} } x_{ij} \le x_{io} \quad i = 1,\,\,2,\, \ldots ,\,m, \\ & \sum\limits_{j = 1}^{n} {\lambda_{j} } y_{rj} \ge \theta_{o} y_{ro} \quad r = 1,\,2,\, \ldots ,\,s, \\ & \sum\limits_{j = 1}^{n} {\lambda_{j} } = 1 \\ & \lambda_{j} \ge 0\quad j = 1,2, \ldots ,n \\ \end{aligned}$$

This model delivers an efficiency score of $\frac{1}{{\theta_{o} }}$ for ${\text{Patient}}_{O}$, with $\theta_{o} \ge 1$. A value of $\theta_{o} > 1$ implies that ${\text{Patient}}_{O}$ underperforms. Given the optimal values of $\theta_{o}^{*}$ just derived, the reference set associated with ${\text{Patient}}_{O}$ is obtained by solving the following model:

2 $$\begin{aligned} & \max \,\,\,\,\sum\limits_{i = 1}^{m} {s_{i}^{ - } } + \sum\limits_{r = 1}^{s} {s_{r}^{ + } } \\ & {\text{subject}}\;{\text{to}} \\ & \sum\limits_{j = 1}^{n} {\lambda_{j} } x_{ij} + s_{i}^{ - } = x_{io} \quad i = 1,\,\,2,\, \ldots ,\,m, \\ & \sum\limits_{j = 1}^{n} {\lambda_{j} } y_{rj} - s_{r}^{ + } = \theta_{o}^{*} y_{ro} \quad r = 1,\,2,\, \ldots ,\,s, \\ & \sum\limits_{j = 1}^{n} {\lambda_{j} } = 1 \\ & \lambda_{j} \ge 0\quad j = 1,2, \ldots ,n, \\ & s_{i}^{ - } \ge 0\quad i = 1,\,\,2,\, \ldots ,\,m, \\ & s_{r}^{ + } \ge 0\quad r = 1,\,\,2,\, \ldots ,\,s. \\ \end{aligned}$$

The convexity constraint defining the variable returns to scale, $\,\sum\nolimits_{j = 1}^{n} {\,\lambda_{j} } = 1$, guarantees that the reference points determining the relative performance of the different patients are composed by weighted averages of well-performing patients. That is, the model generates a benchmark frontier that does not necessarily rely on a particular patient but defines weighted reference values out of several well-performing patients.

##### **Definition**

Let $\theta_{o}^{*}$, $s_{i}^{ - *}$ and $s_{r}^{ + *}$ be the optimal solutions associated with the previous models.

${\text{Patient}}_{O}$ performs well if an optimal solution $\left( {\theta_{o}^{*} ,\lambda_{j}^{*} ,s_{i}^{ - *} ,s_{r}^{ + *} } \right)$ satisfies $\theta_{o}^{*} = 1$, $s_{i}^{ - *} = 0$, $s_{r}^{ + *} = 0.$ Otherwise, ${\text{Patient}}_{O}$ underperforms.

The non-parametric quality of DEA allows us to identify the variables where each patient underperforms as well as their corresponding magnitudes relative to the reference benchmark defined by the best performing patients across all variables. In other words, we do not only focus on the general performance of patients derived from the DEA model described in Eq. (1). We concentrate on the capacity of DEA to identify inefficiencies on a per patient and variable basis. That is, patients may display relatively good performances but exhibit inefficiencies on a subset of inputs. In this regard, the concise implementation of DEA described in Eq. (2) is used to identify the relative performance of each patient on a per variable basis.

The slack variables necessary to generate the relative performance index are inherent to any DEA model, independently of its structural complexity (Santos Arteaga et al. 2020). We are focusing on the generation of an index from a structure where the relative performance of patients in terms of inputs can be used to reflect (and extrapolate) their behavior absent efficiency considerations. In this regard, the basic output oriented version is one of the simplest and more intuitive DEA configurations, though slacks and the subsequent performance indexes can be obtained from any of the extended versions of DEA introduced in the literature (Santos Arteaga et al. 2019).

The papers described at the beginning of this section do not consider the categorization quality of DEA, a particularly useful feature when dealing with small datasets, as the battery of tests performed in the current paper illustrates. It should be highlighted that in our analysis, all patients but one behave efficiently, that is, display a $\theta_{o}^{*}$ value equal to one. Clearly, this information is irrelevant to the physicians, whose ANN models remain unable to identify the main characteristics of the patients displaying the worst potential behavior.

#### Generating the performance index

The output-oriented DEA model delivers a matrix describing both the efficiency of patients as well as the value of the slacks for each input and output variable. Both the efficiencies and the slacks allow to categorize patients in clusters determined by their relative performances. We do not focus on the value of $\theta_{o}^{*}$ displayed by the patients but generate a relative performance index based on the value of the input slack variables.

In this respect, our approach diverges considerably from that of a standard operational research environment. The main objective of a DEA model is to identify the most efficient patients in the sample, though in the current context this information is not particularly useful to physicians. The behavior of patients can however be evaluated through the value of the slack variables, a feature that will be used to profile the relative performance of the patients.

DEA considers a matrix consisting of 17 input and 3 output variables per patient to compute the corresponding efficiency scores while also generating a profile for each patient consisting of 20 slack values. We have normalized the slack values obtained from DEA to define a relative performance profile of the patients in terms of the initial values displayed by the variables. In this case, higher values are associated with relatively worse evaluations.

We abuse notation and use $s_{ij}^{ - }$, $j = 1,\,\,2,\, \ldots ,\,n,\,\,i = 1,\,\,2,\, \ldots ,\,m$, in the normalization equations below to refer to the slack variables obtained from the model described in Eq. (2),

3 $$s_{i}^{ - } = x_{io\,} - \sum\limits_{j = 1}^{n} {\,\lambda_{j} } x_{ij\,} ,\quad i = 1,\,\,2,\, \ldots ,\,m,$$

which define the distance between the variables composing the profile of each patient and a convex combination of benchmark reference values.

The slacks of quantitative variables have been normalized with respect to the initial values displayed by the patient following a standard approach

4 $$\tilde{x}_{ij} = \frac{{s_{ij}^{ - } }}{{x_{ij\,} }},\,\,\,\,\,\,\,\,\,j = 1,\,\,2,\, \ldots ,\,n;\quad i = 1,\,\,2,\, \ldots ,\,m.$$

Note that the farther the distance from the optimal input level displayed by a given patient $j$ in terms of variable $i$, the higher the relative value of the normalized slack and the poorer the performance of the patient. The idea is indeed quite simple, patients must improve with respect to their initial analysis values, allowing for a common general comparison framework.

Categorical values follow a similar intuition in terms of normalization relative to the initial value of the category to which the patient belongs. The normalization of the categorical variables requires two reference values, namely, the highest value of the category, $\max_{j} \left\{ {x_{ij\,} } \right\}$, and the original value of the variable exhibited by patients

5 $$\hat{x}_{ij} = \frac{{s_{ij}^{ - } + x_{ij\,} }}{{x_{ij\,} + \max_{j} \left\{ {x_{ij\,} } \right\}}},\quad j = 1,\,\,2,\, \ldots ,\,n;\,\,\,i = 1,\,\,2,\, \ldots ,\,m.$$

Clearly, the highest value of an input category differs across variables according to the potential categories defined per variable. For instance, the maximum values of the categories defined for the following variables are: diabetes mellitus = 1, hypertension = 1, ACEI/ARB = 1, cough as presenting symptom = 1, acute kidney injury = 1, and pneumonia = 2.

The index generated using the normalized slack values is defined as follows. We start by adding up the value of all the normalized slacks for each patient

6 $$x_{j}^{s} = \sum\limits_{i = 1}^{m} {\tilde{x}_{ij} + \sum\limits_{i = 1}^{m} {\hat{x}_{ij} } } ,\quad j = 1,\,\,2,\, \ldots ,n.$$

Define the highest value of the sum of the normalized slacks as follows

7 $$x^{m} = \max \left\{ {x_{j}^{s} ,\,\,\,j = 1,\,\,2,\, \ldots ,\,n} \right\}.$$

The value of the relative slack-based performance index assigned to each patient is therefore given by

8 $$p_{j} = \frac{{x_{j}^{s} }}{{x^{m} }},\quad j = 1,\,\,2,\, \ldots ,n.$$

Patients are then classified in quartile or tercile categories depending on their relative performances as determined by $p_{j}$. We should note that the relatively low number of observations and the fact that most patients performed considerably well has led to a very small number of patients being categorized within the worst quartiles or terciles.

Figure 3a, b illustrate the capacity of the normalized slacks values to categorize patients according to their relative performance. We have used red circles to represent the initial value of the variables displayed by the patients. Similarly, blue circles describe the value of the normalized slack variables derived from DEA. The planes dividing the space are conditioned by the initial domains defining the value of the variables. The capacity of the normalized slacks to identify clusters of patients based on their relative performance on a per variable and patient basis can be observed in both figures.

#### Categorization process

The categorization of each alternative configuration is summarized in Table S5. The slack-based index generated using DEA distributes patients across three categories. A few of them display a normalized index value within [1, 0.75], indicating a suboptimal performance categorized as the worst quartile. Most of the patients perform above average, that is, the normalized values of their indexes are contained within the interval [0.5, 0], which has been divided in the corresponding third and best quartiles. Given the low number of observations and the relatively good outcomes observed among patients, the index does not deliver any value within the interval [0.75, 0.5].

We must emphasize that these numerical results are conditioned by the small size of the sample with which we are initially endowed. The implementation of DEA encompasses a total of 29 patients, namely, those displaying observations for all the input and output variables considered. Despite the severity of the pandemic, the initial number of kidney transplant recipients affected by the virus was small. The capacity of DEA to generate a performance profile of patients with a small number of observations constitutes one of its main qualities and advantages over standard statistical methods. However, the number of categories generated depends on the number of patients that can be assigned to each one of them. With relatively few observations, we must consider adapting the categories to the results obtained, a drawback that can be accounted for with relatively larger amounts of patients.

In order to prevent selection biases, the output categories used to train the ANN have been defined to contain all the potential distributions of patients. In particular, we consider three different configurations of the output variables to improve upon the results of the DEA hybrid. The three alternative configurations have been defined based on the actual evolution of the patients. The first configuration, CNF1, distributes patients in three different categories as follows: if patients require all three output treatments, they are included within the worst category. Patients requiring one and two output treatments comprise the second category, while those not requiring any treatment constitute the best performing category. The distribution of patients across categories inherent to this configuration is the closest one to that of the DEA hybrid model. The second configuration, CNF2, considers patients requiring three and two output treatments as those composing the worst category. Patients requiring one treatment were included within the second category, while those not requiring any treatment comprised the best performing category. The third and final configuration, CNF3, defines a total of four categories, one for each number of treatments being required.

We must finally note that several modifications have been applied when defining the logistic regression, requiring a unique binary category to represent the dependent variables. Two configurations of the hybrid model have been defined. The first DEA configuration assigns a value of one to the worst quartile and a value of zero to the remaining ones. The second DEA configuration assigns a value of one to the worst and third quartiles and a value of zero to the best quartile (though not reported in Table 3, the accuracy achieved by the second DEA configuration equals 85.2%). The same intuition applies to the logistic categorization of the alternative configurations. The corresponding assignment of binary variables to the hybrid model and each alternative configuration is described in Table S6.

#### Comparative technical analysis

Selection biases have been prevented by considering all the potential distributions of patients across the output categories used to train the ANN. In addition, the accuracy of these alternative configurations relative to that of the hybrid model has been tested through a battery of 24 machine learning techniques, including logistic regression and random forest. Detailed descriptions of all these techniques, together with their advantages and drawbacks, can be found in any textbook on machine learning (Bishop 2006). We briefly summarize each one of them in the next section. All the numerical simulations have been performed using MATLAB software.

#### Supervised machine learning techniques implemented

We provide a brief description of the supervised machine learning techniques applied to illustrate the identification enhancing quality of the DEA-generated index relative to a direct implementation of the techniques.

Intuitively speaking, when facing a classification problem, we are endowed with a set of predictors, namely, features or independent variables, and a given outcome, that is, a class or dependent variable. Each observation consists of a set of predictor values and the corresponding class. The techniques described learn from the set of predictor values and their corresponding classes so that whenever a given set of feature values is received, the technique can predict the class to which the object belongs.

##### Artificial neural network

An artificial neural network is composed by an input layer, one or more hidden layers, and an output layer. Each layer consists of several nodes, i.e., neurons, interconnecting the layers. The network is based on a sequential process where layer inputs are given by the output of the previous layer. The strength of the signals issued by the neurons is determined by the adjustment of their weights taking place through the learning process. ANNs aim at recognizing patterns so as to classify inputs into different classes according to key characteristics via either supervised or unsupervised learning.

##### Logistic regression

Logistic regression is one of the classification algorithms most commonly used when the objects analyzed must be categorized in two main classes. The classifier determines the probability of belonging to each class combining linearly a given set of predictors.

##### Decision trees

Decision trees allow to classify objects into a finite number of classes determined by a categorical target attribute. A hierarchical tree structure consists of a set of nodes based on attributes that split into branches determined by the potential values of the attributes, leading to the final leaf nodes encompassing the membership classes of the target attribute. Trees may target a single attribute or a composed one based on a linear combination of variables.

The main types of decision trees are determined by the number of leaves defined to distinguish among classes, ranging from fine (allowing for a maximum of 100 node splits), and medium (endowed with a maximum of 20 node splits) to coarse (where the maximum number of node splits equals 4).

##### Random forest

A random forest consists of multiple individual decision trees operating as an ensemble. Trees deliver class predictions and select the class with the highest number of votes. Correlation across trees is prevented through bagging (bootstrap aggregation)—such that each tree samples randomly with replacement from the dataset –, and feature randomness—such that trees choose from a random subset of features when selecting node branches –.

##### Linear discriminant

Linear discriminant is a classification technique that defines the main differences between a set of classes using linear boundaries. Classes are assumed to generate data through different Gaussian distributions, with fitting functions estimating the corresponding parameters for each class. The characteristics of the objects are used to determine the class where they are allocated, with each object receiving scores from the different predictors and a group one.

##### Kernel Naïve bayes

The naïve Bayes classifier consists of two main steps. The first one is training: the classifier estimates the parameters of a probability distribution assuming the conditional independence of predictors given the class. More precisely, the classifier assumes that the effects that predictors have on each class are independent of the values of the other predictors. The second step is prediction: the classifier computes the posterior probability of a new data sample belonging to each class, with the data being classified according to the highest probability.

##### Support vector machine

Support vector machine defines a hyperplane to separate objects across classes. This technique aims to find the hyperplane with the widest margin between classes. The support vectors are given by the points located closest to the hyperplane. The categorization process depends on the type of classifier used to separate the classes, which ranges from linear, quadratic, and cubic functions to Gaussian kernels of different scales—fine, medium, and coarse –.

##### K-nearest neighbors

The k-nearest neighbors classifier categorizes objects according to their distance to other neighboring objects within the dataset. Depending on the degree of distinction between classes, namely, the number of neighboring points considered, we have different versions of the classifier ranging from fine (one neighbor) and medium (10 neighbors) to coarse (100 neighbors). In addition, MATLAB allows to implement cosine and cubic metrics, as well as distance weights, while considering 10 neighbors.

##### Ensemble

Ensemble classifiers define hybrid techniques incorporating the output from different weak learners into a higher quality model. We implement the following classifiers in our analysis.

- *Boosted Trees* combining Adaptive Boost with Decision Tree learners;
- *Bagged Trees* combining a Random Forest Bag with Decision Tree learners;
- *Subspace Discriminant* combining Subspace classifiers with Discriminant learners;
- *Subspace KNN* combining Subspace classifiers with Nearest Neighbor learners;
- *RUSBoosted Trees* combining Random Under-Sampling Boost with Decision Tree learners.

#### Death and days spent in intensive care as outputs

We conclude by performing an additional set of simulations comparing the outcomes obtained from the direct implementation of the ANN with those derived from the slack-based DEA-ANN. In this case, the output variables considered are death and number of days spent in intensive care. These variables were not part of the composite primary outcome analyzed by the physicians but are particularly important during the early stages of a medical emergency. We have analyzed these variables separately since their incorporation into the analysis would require a much more complex combinatorial scenario to define the potential output configurations of the ANN. On the other hand, we should emphasize that these variables can be easily incorporated into the slack-based index defined through DEA.

Given the heterogenous values displayed by the number of days in an ICU, we have categorized the variable by considering whether patients spent more or less than two weeks in intensive care. That is, we are endowed with a binary output variable representing death and an additional categorical variable describing whether patients spent more or less than two weeks, or no time at all, in an ICU. The division of these outputs across the classes defined to train the ANN and implement the slack-based DEA index is presented in Table S7. We have considered all possible categorization strategies designed to compare the identification capacities of the ANN and the hybrid DEA-ANN technique.

The corresponding set of confusion matrices obtained within each setting is presented in Figures S1 to S3. Note how, in all settings, the ANN exhibits substantial problems identifying the patients who end up needing intensive care or dying. Indeed, the identification capacity of the ANN relies on those patients who do not die or require any time in an ICU. The enhanced DEA-ANN model overcomes this identification problem when defining the relative performance of each patient. As can be observed, its identification capacity improves substantially upon that of the ANN. Intuitively, DEA considers the performance of each patient relative to the whole population being analyzed, which implies that patients whose analyses display poor results and end up in an ICU are not necessarily defined as inefficient. On the other hand, patients displaying regular values in their analyses but ending in an ICU are the ones who may be considered inefficient.

We conclude by illustrating the slack-based performance profiles of patients categorized by terciles in Figure S4. It can be immediately observed that the profiles generated by the slacks are quite similar to those obtained when considering the composite primary outcome variables as the outputs of the DEA-ANN model. Thus, besides (generally) improving the identification capacity of the corresponding machine learning techniques, DEA allows us to generate consistent profiles of the patients determined by their potential evolution.

## Code availability statement

The MATLAB code used in this study is available from the corresponding author (F.J.S.A.) upon reasonable request.
