# Supplementary material for: A hybrid data envelopment analysis—artificial neural network prediction model for COVID-19 severity in transplant recipients
Source: Artif Intell Rev. 2021 Apr 23;54(6):4653–84. doi: 10.1007/s10462-021-10008-0 (PMC8062617; doi:10.1007/s10462-021-10008-0)
Supplement: Supplementary file 1 — Supplementary file1 (DOCX 237 kb) [file 10462_2021_10008_MOESM1_ESM.docx]

**Supplementary Figures Appendix**

**Figure S1.** Quartile Setting

**Figure S1(a).** ANN configuration

**Figure S1(b).** DEA-ANN configuration

**Figure S2.** Tercile Setting

**Figure S2(a).** First ANN configuration: CNF1

**Figure S2(b).** Second ANN configuration: CNF2

**Figure S2(c).** DEA-ANN configuration

**Figure S3.** Median Setting

**Figure S3(a).** ANN configuration

**Figure S3(b).** DEA-ANN configuration

**Figure S4.** Patient performance across input variables for the different index categories generated using DEA when death and days spent in intensive care are taken as outputs


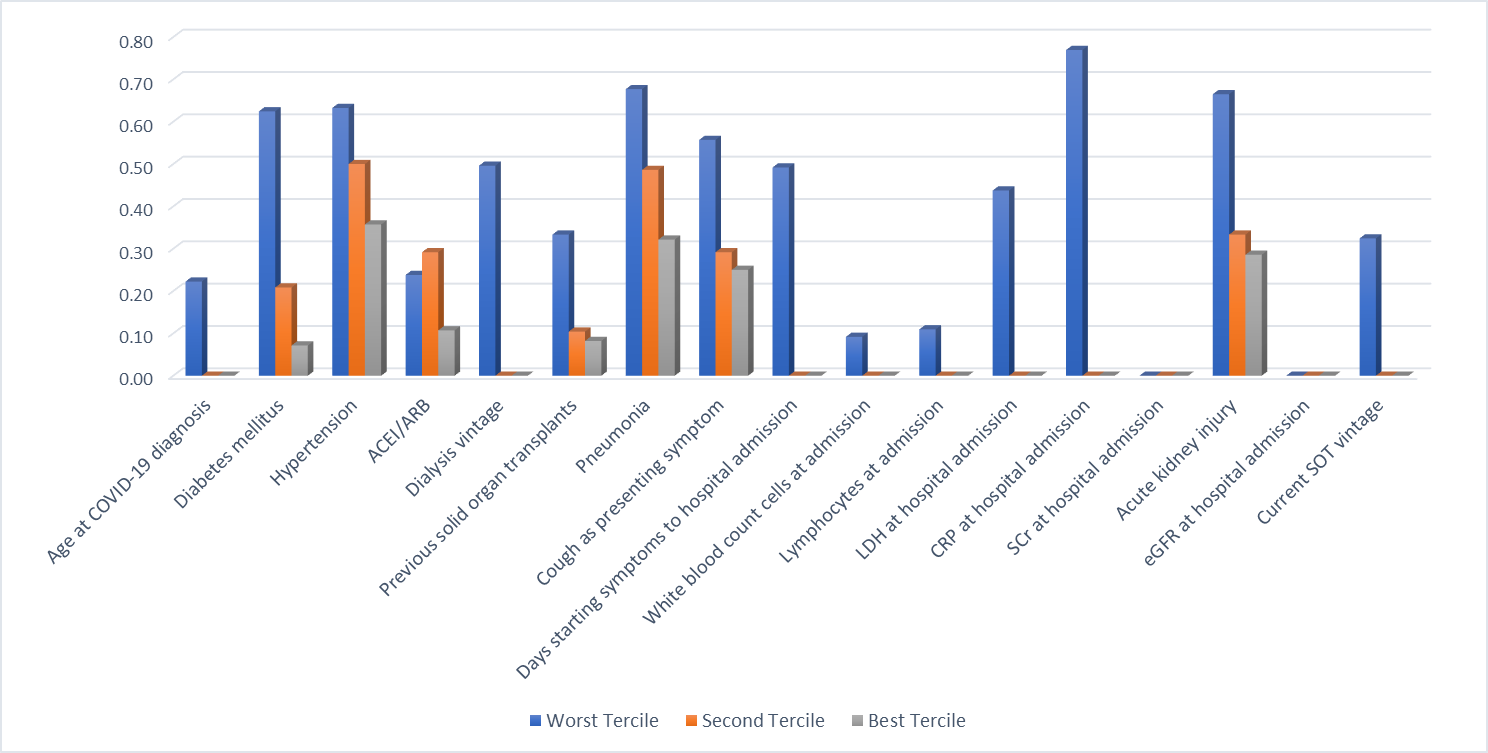


**Legend:** The index has been normalized within the interval [0, 1]. Higher values represent relatively worse performances.

**Supplementary Tables Appendix**

**Extended Data Table S1.** Kidney and transplant characteristics

| Etiology of Chronic Kidney Disease, n (%) | |
| --- | --- |
| Glomerulonephritis | 9 (23.7) |
| Polycystic disease | 8 (21.1) |
| Diabetic nephropathy | 6 (15.8) |
| Hypertensive nephroangiosclerosis | 5 (13.2) |
| Unknown | 5 (13.2) |
| Others | 5 (13.2) |
| Type of transplant, n (%) | |
| Kidney transplant | 35 (92.1) |
| Multiple organ transplant | 3 (7.9) |
| Source of donor, n (%) | |
| Decease donor | 30 (78.9) |
| Living donor | 8 (21.1) |
| Other comorbidities, n (%) | |
| Dyslipidemia | 14 (36.8) |
| Coronary Artery Disease | 4 (10.5) |
| Immunosuppressive drug at COVID-19 diagnosis, n (%) | |
| Calcineurin inhibitor | 33 (86.8) |
| Antimetabolite drug | 24 (57.9) |
| mTOR inhibitor | 16 (42.1) |
| Prednisone | 32 (84.2) |

**Extended Data Table S2.** Hospital admission laboratory results

| Mean serum creatinine at hospital admission (mg/dl) (s.d.) (range) | 1.59 (0.65) (0.7-3.4) |
| --- | --- |
| Mean eGFR at hospital admission (ml/min/1.73 m2) (s.d.) (range) | 49.47 (19.17) (18-95) |
| Mean white cell count at hospital admission (s.d.) (range) | 6734 (3154) (2800-13400) |
| Mean lymphocytes at hospital admission (s.d.) (range) | 877 (1419) (0-8700) |
| Mean haemoglobin at hospital admission (g/dL) (s.d.) (range) | 12.49 (2.07) (8.8-17.5) |
| Mean platelets at hospital admission (s.d.) (range) | 192969 (77687) (71000-404000) |
| Mean AST at hospital admission (IU/L) (s.d.) (range) | 42.37 (32.01) (14-142) |
| Mean ALT at hospital admission (IU/L) (s.d.) (range) | 34.63 (26.74) (8-127) |
| Mean GGT at hospital admission (IU/L) (s.d.) (range) | 77.77 (67.24) (13-378) |
| Mean LDH at hospital admission (IU/L) (s.d.) (range) | 310.26 (107.92) (142-529) |
| Mean CRP at hospital admission (mg/dL) (s.d.) (range) | 13.58 (7.78) (0.4-32) |
| Mean procalcitonin at hospital admission (ng/mL) (s.d.) (range) | 0.42 (0.57) (0-1.9) |
| Mean ferritin at hospital admission (ng/mL) (s.d.) (range) | 513.50 (422.78) (70-1566) |
| Mean D-dimer at hospital admission (ng/mL) (s.d.) (range) | 1316.13 (1339.03) (200-5600) |
| Mean high sensitivity Troponin I at hospital admission (ng/mL) (s.d.) (range) | 58.85 (168.91) (3.8-803) |

**Acronyms:** eGFR: estimated Glomerular Filtrate Rate; AST: Aspartate transaminase; ALT: Alanine transaminase; GGT: gamma glutamyl transferase; LDH: Lactate dehydrogenase; CRP: C-reactive protein.

**Extended Data Table S3.** ICU comparative analysis

| **Variable** | **Intensive Care Unit (N=9)** | **Non-intensive Care Unit (N=29)** | **P value** |
| --- | --- | --- | --- |
| Cough as presenting symptom, n (%) | 8 (88.9) | 15 (51.7) | 0.030 |
| Pneumonia, n (%) |  |  |  |
| No | 0 (0.0) | 7 (24.1) | 0.032 |
| Unilateral | 0 (0.0) | 7 (24.1) |  |
| Bilateral | 9 (100.0) | 15 (51.8) |  |
| Mean lactate dehydrogenase (IU/L) (s.d.) | 397.86 (130.41) | 281.47 (84.54) | 0.009 |
| Mean CRP (mg/dl) (s.d.) | 11.99 (4.73) | 8.76 (7.94) | 0.011 |
| Mean D-dimer (ng/mL) (s.d.) | 5000 (4687.93) | 2113.45 (1776.83) | 0.021 |
| Steroid pulse, n (%) | 7 (77,8) | 10 (34.5) | 0.034 |
| Mean days at hospital(s.d.) | 16.33 (7.19) | 10.48 (6.11) | 0.026 |
| Graft loss, n (%) | 2 (22.2) | 4 (13.8) | 0.457 |
| Death, n (%) | 2 (22.2) | 3 (10.3) | 0.352 |

Results include all recipients who were hospitalized with COVID-19 from March 3 to April 24, and followed-up till April 27,2020.

χ2 test (or Fisher’s exact test whenever appropriate), Student’s t test, and analysis of variance (Mann-Whitney test).

**Table S4.** Input profiles of patients and output treatment variables

| **Input Variables** | **Output Variables** |
| --- | --- |
| Age at COVID-19 diagnosis | ICU admission |
| Diabetes mellitus | Treatments with tocilizumab |
| Hypertension | Pulses of methylprednisolone |
| Angiotensin-converting-enzyme inhibitors (ACEI) / angiotensin-receptor blockers (ARB) treatment |  |
| Dialysis vintage (months) |  |
| Previous SOT |  |
| Pneumonia |  |
| Cough as presenting symptom |  |
| Time from starting symptoms to hospital admission (days) |  |
| White blood count cells at admission |  |
| Lymphocytes at admission |  |
| LDH at hospital admission |  |
| CRP at hospital admission |  |
| SCr at hospital admission |  |
| Acute kidney injury |  |
| eGFR at hospital admission |  |
| Current SOT vintage |  |

**Table S5.** Categorization of the index values and each alternative configuration

|  | **Index Values and Categories** | | | |
| --- | --- | --- | --- | --- |
|  | [1, 0.75] | [0.75, 0.5] | [0.5, 0.25] | [0.25, 0] |
| **DEA** | Worst Quartile | - | Third Quartile | Best Quartile |
|  | **Number of Output Treatments per Quartile** | | | |
| **CNF1** | 3 | - | 2 and 1 | 0 |
| **CNF2** | 3 and 2 | - | 1 | 0 |
| **CNF3** | 3 | 2 | 1 | 0 |

**Table S6.** Binary variables assigned to the hybrid model and each alternative configuration

|  | **Index Values and Logistic Categories** | | | |
| --- | --- | --- | --- | --- |
|  | [1, 0.75] | [0.75, 0.5] | [0.5, 0.25] | [0.25, 0] |
| **DEA** | 1 | - | 0 | 0 |
| **DEA2** | 1 | - | 1 | 0 |
|  | **Number of Output Treatments per Logistic Category** | | | |
| **CNF1**  **Logistic** | 3  1 | - | 2 and 1  0 | 0  0 |
| **CNF2**  **Logistic** | 3 and 2  1 | - | 1  0 | 0  0 |
| **CNF3**  **Logistic** | 3  1 | 2  1 | 1  1 | 0  0 |

**Table S7.** Categorization of the index values and the alternative configurations when death and days spent in intensive care are taken as outputs

**Table S7(a).** Quartile setting

|  | **Index Values and Categories** | | | |
| --- | --- | --- | --- | --- |
|  | [1, 0.75] | [0.75, 0.5] | [0.5, 0.25] | [0.25, 0] |
| **DEA** | Worst Quartile | Second Quartile | Third Quartile | Best Quartile |
|  | **Outputs per Category** | | | |
| **CNF** | Death | ICU > 2 weeks | ICU < 2 weeks | None |
| **Class** | 1 | 2 | 3 | 4 |

**Table S7(b).** Tercile setting

|  | **Index Values and Categories** | | |
| --- | --- | --- | --- |
|  | [1, 0.67] | [0.67, 0.33] | [0.33, 0] |
| **DEA** | Worst Tercile | Second Tercile | Best Tercile |
|  | **Outputs per Category** | | |
| **CNF1** | Death | ICU | None |
| **CNF2** | Death and ICU > 2 weeks | ICU < 2 weeks | None |
| **Class** | 1 | 2 | 3 |

**Table S7(c).** Median setting

|  | **Index Values and Categories** | |
| --- | --- | --- |
|  | [1, 0.5] | [0.5, 0] |
| **DEA** | Worst Median | Best Median |
|  | **Outputs per Category** | |
| **CNF** | Death and ICU | None |
| **Class** | 1 | 2 |
